# Conservation genomics of *Agave tequilana* Weber var. azul: low genetic differentiation and heterozygote excess in the tequila agave from Jalisco, Mexico

**DOI:** 10.7717/peerj.14398

**Published:** 2022-11-17

**Authors:** Karen Yazmin Ruiz Mondragon, Erika Aguirre-Planter, Jaime Gasca-Pineda, Anastasia Klimova, Roberto-Emiliano Trejo-Salazar, Marco Antonio Reyes Guerra, Rodrigo A. Medellin, Daniel Piñero, Rafael Lira, Luis E. Eguiarte

**Affiliations:** 1 Instituto de Ecología, Universidad Nacional Autónoma de México, Ciudad de México, Mexico; 2 Laboratorio de Recursos Naturales, Unidad de Biotecnología y Prototipos (UBIPRO), Facultad de Estudios Superiores Iztacala, Universidad Nacional Autónoma de México, Ciudad de México, México

**Keywords:** Clonality, Gene flow, Genetic resources, Genomic diversity, Inbreeding, Single nucleotide polymorphisms (SNPs)

## Abstract

**Background:**

Genetic diversity is fundamental for the survival of species. In particular, in a climate change scenario, it is crucial that populations maintain genetic diversity so they can adapt to novel environmental conditions. Genetic diversity in wild agaves is usually high, with low genetic differentiation among populations, in part maintained by the agave pollinators such as the nectarivorous bats. In cultivated agaves, patterns of genetic diversity vary according to the intensity of use, management, and domestication stage. In A*gave tequilana* Weber var. azul (*A. tequilana* thereafter), the plant used for tequila production, clonal propagation has been strongly encouraged. These practices may lead to a reduction in genetic diversity.

**Methods:**

We studied the diversity patterns with genome-wide SNPs, using restriction site associated DNA sequencing in cultivated samples of *A. tequilana* from three sites of Jalisco, Mexico. For one locality, seeds were collected and germinated in a greenhouse. We compared the genomic diversity, levels of inbreeding, genetic differentiation, and connectivity among studied sites and between adults and juvenile plants.

**Results:**

*Agave tequilana* presented a genomic diversity of *H_T_* = 0.12. The observed heterozygosity was higher than the expected heterozygosity. Adults were more heterozygous than juveniles. This could be a consequence of heterosis or hybrid vigor. We found a shallow genetic structure (average paired *F_ST_* = 0.0044). In the analysis of recent gene flow, we estimated an average migration rate among the different populations of *m* = 0.25. In particular, we found a population that was the primary source of gene flow and had greater genomic diversity (*H_E_* and *H_O_*), so we propose that this population should continue to be monitored as a potential genetic reservoir.

**Discussion:**

Our results may be the consequence of more traditional management in the studied specific region of Jalisco. Also, the exchange of seeds or propagules by producers and the existence of gene flow due to occasional sexual reproduction may play an important role in maintaining diversity in *A. tequilana*. For populations to resist pests, to continue evolving and reduce their risk of extinction under a climate change scenario, it is necessary to maintain genetic diversity. Under this premise we encourage to continue acting in conservation programs for this species and its pollinators.

## Introduction

Conservation genetics combines evolutionary theory and molecular markers to help biodiversity conservation (Frankham, 2010). An important component of this discipline is to understand how genetic diversity is generated and maintained (Eguiarte & Souza, 2007). Genetic diversity is fundamental for the survival of species and populations (Bhandari et al., 2017), particularly in a changing environment (Frankham, 2010). It is well known that a reduction of genetic diversity is generally associated with a fitness reduction, diminished evolutionary potential, and an increased risk of extinction (Gepts & Hancock, 2006; Frankham, 2010; Bruford et al., 2017).

In crop plants, the levels of genetic diversity contained in the managed and in the wild (if still extant) gene pools are relevant to further crop improvement and as a source of resistance to diseases and adaptation to the changing climate (Gepts & Papa, 2003). However, in cultivated plants this diversity may decrease at accelerated rates, due to the replacement of traditional varieties with uniform, high-yield crops, that are usually monocultured (Millennium Ecosystem Assessment, 2005; FAO, 2010; Bruford et al., 2017). As a result, these plants may be susceptible to environmental change, pests, and diseases (Bruford et al., 2017). Therefore, to avoid genetic erosion and prevent the loss of alleles through selective breeding, it is essential to gather information on the patterns of genetic variation in plant species under management, as well as their wild relatives. In addition, knowledge of population structure and relationships within and between wild and cultivated populations is crucial in supporting modern breeding programs.

One interesting example of how modern breeding programs may affect the genetic structure and diversity of a crop species is *Agave tequilana* Weber var. azul (*A. tequilana* hereafter). *A tequilana* is a diploid species (2n = 2x = 60), with a genome size of 3,677.45 Mbp (Robert et al., 2008). Like other agave species, it can combine sexual and vegetative (aerial bulbils and ground-level basal shoots and rhizomes) reproduction (Eguiarte et al., 2013). This species is used for tequila production and has enormous economic relevance for Mexico. Tequila production from *A. tequilana* started in the nineteenth century (Colunga-García Marín & Zizumbo-Villarreal, 2007); the preference to use this species is because it matures relatively fast, around eight years, and also to its ability to accumulate high levels of fructans in comparison to other agave species (Trejo et al., 2018). The high demand for tequila has encouraged intensive management and clonal propagation of the plants (Dalton, 2005).

Clonal reproduction in crop species is not uncommon, at least 34 plant families present it, including herbs, shrubs, trees and vines, such as cassava (*Manihot esculenta*), taro (*Colocasia esculenta* L.), potato (*Solanum tuberosum*), grapevines (*Vitis vinifera*), strawberry (*Fragaria spp*.) and so on (McKey et al., 2010), as it has several advantages, such as maintaining valuable traits and ease of propagation (McKey et al., 2010). Clonal propagation in these outcrossing plants helps to preserve heterozygous genotypes that show hybrid vigor (Dobzhansky, 1952; Balloux, Lehmann & de Meeüs, 2003; Glémin, Bazin & Charlesworth, 2006). Nevertheless, clonal propagation may also lead to genetic erosion, the spread of pathogens, and the accumulation of deleterious mutations (McKey et al., 2010).

However, the consequences of clonal propagation on the genetic diversity of *A. tequilana* are still not clear. Some authors reported no genetic diversity (Gil-Vega et al., 2001; Trejo et al., 2018), which may make the species particularly vulnerable to pathogens (Gil-Vega et al., 2001; Dalton, 2005). Other studies have suggested a less pronounced reduction of the genetic variation (Gil-Vega et al., 2006; Vargas-Ponce et al., 2009; Rivera-Lugo, García-Mendoza & Simpson, 2018; Cabrera-Toledo et al., 2022). This discrepancy may result from the variation of the marker used, study design, as well as management intensity of the sampled populations.

Genetic diversity in cultivated agaves varies according to the intensity of use, management, and domestication (Eguiarte et al., 2013, 2021; Trejo et al., 2018; Álvarez-Ríos et al., 2020; Klimova et al., 2022). In wild agaves, genetic diversity is usually high, with low genetic differentiation among populations (see reviews in Eguiarte et al., 2013, 2021; Klimova et al., 2022), a pattern that is maintained in part by the most important agave pollinators, such as the nectarivorous bats, *Leptonycteris nivalis* and *L. yerbabuenae* (Eguiarte, Souza & Silva-Montellano, 2000; Rocha et al., 2006; Trejo-Salazar, Scheinvar & Eguiarte, 2015; Eguiarte et al., 2021). Recent conservation and management initiatives have focused on preserving the natural agave pollinators (*e.g*., bats) and, at the same time, mitigating genomic erosion and promoting sustainable practices of the agroecosystems where the main crop is agave used for mezcal, tequila, and other agave distillates production (Trejo-Salazar et al., 2016; see also https://batfriendly.org/).

The recent advent of reduced representation genomic strategies that allows the analysis of many individuals and thousands of single nucleotide polymorphisms (SNPs) has revolutionized studies on the genetic diversity in plant and animal species (Barrera-Redondo, Piñero & Eguiarte, 2020; Eguiarte et al., 2022). This type of markers allows to perform a more precise analysis of the micro-evolutionary processes that occur in the species and the exploration of diversity throughout the entire genome and was recently successfully used in a close relative of *A. tequilana, A. angustifolia* (Cabrera-Toledo et al., 2020; Klimova et al., 2022). We believe that information on genome-wide patterns of genetic variation and knowledge of the population structure of *A. tequilana* will be essential in defining management priorities, developing new sustainable cropping systems, and understanding the impact of domestication on its genetic repertoire.

In this research, we studied genomic diversity patterns in *A. tequilana* collected in Jalisco, using SNPs derived from Restriction-site associated DNA sequencing, or RADseq methodology (Davey & Blaxter, 2010). We compared the diversity in adult and juvenile plants, evaluating levels of inbreeding, genetic differentiation, and connectivity among studied sites. Due to the intense management and mainly clonal reproduction of the species, where the plants are seldom allowed to produce fruits, we expected little genomic diversity and a shallow population structure with low connectivity. Therefore, we aimed to determine if the genomic diversity was reduced in these populations and whether their ability to adapt has been compromised.

## Materials and Methods

### Plant material

**Figure 1:**
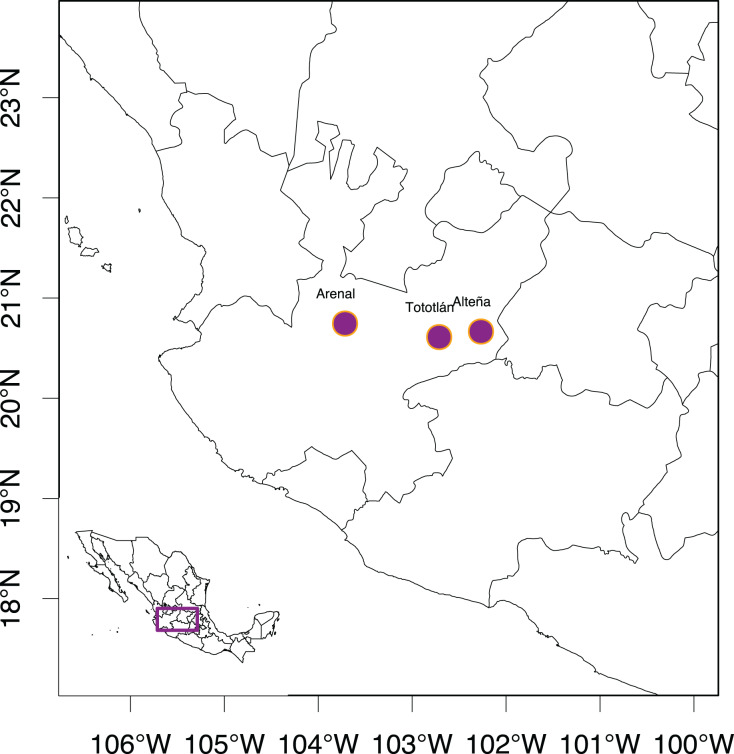
Studied localities of *Agave tequilana* in the state of Jalisco, México.

Plant material was collected from individuals 10 m apart from each other (to avoid clonality) from three “Bat-friendly” plantations, separated by ~90 to 250 km, of *A. tequilana* in Jalisco, Mexico (Table 1 and Fig. 1). In these plantations, tequila production is less intensive, based on more rustic/traditional methods in comparison to the production of tequila in the lower lands of Jalisco, around the town of Tequila. In the studied crop, 5% of the total individuals in the plantations were allowed to blossom to produce nectar for their pollinators, particularly for the bats of the genus *Leptonycteris* (Trejo-Salazar et al., 2016; https://batfriendly.org/).

**Table 1:** *Agave tequilana* collections from Jalisco, Mexico.

ID	Location	Latitude	Longitude	Elevation above sea level (m)	Collection year	*N*
Alteña	Arandas-I	20.6658667	−102.2673722	2,143	2016	11
Alteña	Arandas-II	20.6658667	−102.2673722	2,143	2017	27
Arenal	El Arenal-I	20.7454083	−103.7146194	1,389	2016	10
Arenal	El Arenal-II	20.7454083	−103.7146194	1,389	2017	10
Tototlán	Tototlán	20.6102611	−102.7125444	1,758	2016	10
J-Tototlán	Tototlán	20.6102611	−102.7125444	1,758	2018	28

**Note:**

Number of individuals (*N*) by location and year of collection. For the village of Tototlán, seeds (ID: J-Tototlán) were germinated in the greenhouse of the Instituto de Ecology, Universidad Nacional Autónoma de Mexico.

We analyzed 96 samples collected in two consecutive years (2016 and 2017) from three “Bat-friendly” localities (Table 1 and Fig. 1). From these 96 samples, 68 individuals were mature plants assigned in the “adult” category ca. 6–7 years old. In one of the localities (Tototlán), seeds were collected from different inflorescences, from which 28 seeds randomly selected were germinated in the greenhouse of the Instituto de Ecología, Universidad Nacional Autónoma de México (UNAM), in Mexico City, that we will call “juveniles” (less than two years old and non-reproductive). Upon collection, all samples were preserved at −80 °C until DNA extraction.

### DNA extraction and sequencing

For all the samples, genomic DNA was extracted from leaf tissue using a modified “Mini-Prep” CTAB protocol (Doyle & Doyle, 1987; Klimova et al., 2022). DNA was visualized on a 1% agarose electrophoresis gel, and quantified using the Qubit 3.0 fluorometer with Qubit dsDNA broad-range kit and NanoDrop Lite Spectrophotometer by Thermo Fisher Scientific. Libraries preparation and sequencing were performed at the Biotechnology Center of the University of Wisconsin-Madison (https://biotech.wisc.edu/). Each sample was digested using two methylation-sensitive restriction enzymes (*Pst*I and *Msp*I); the choice of enzymes was based on the previous standardization for *Agave salmiana* and *A. lechuguilla* (Dr. Alejandra Moreno-Letelier, Instituto de Biología, UNAM, 2020, personal communication). After specific barcodes were ligated to each sample, those were pooled in equimolar concentration and sequenced using the Illumina NovaSeq 2 × 150 platform (Illumina, Inc., San Diego, CA, USA).

### Bioinformatics analysis

Massive parallel sequencing platforms generate tens of millions of sequences. However, it is essential to verify the quality of these sequences so as not to cause bias in the data analysis. For quality filtering, we first used TRIMMOMATIC (Bolger, Lohse & Usadel, 2014); we removed adapters and low-quality bases using the following parameters: ILLUMINACLIP (Nextera PE-PE.fa: 2:30:10), SLIDINGWINDOW: 4:20, LEADING: 25, TRAILING: 25 and MINLEN: 60.

With the paired files generated with TRIMMOMATIC, we used the reference transcriptome—because there is no published agave genome—of *Agave tequilana* (GAHU00000000.1; Gross et al., 2013). For SNP calling we used the ipyrad software (Eaton & Overcast, 2020), using the option for paired-end data, digested with two enzymes (https://ipyrad.readthedocs.io/).

The final data filtering was performed with VCFtools v.0.1.15 (Danecek et al., 2011); we avoided SNPs from the same locus by using *thin* (100 sites), so that no two sites were within the specified distance from one another, and we also removed SNPs, that significantly deviated from Hardy-Weinberg equilibrium test (*—hwe* 0.000005). We only retained sites with a mean minimum depth of over 12, and maximum two alleles with no InDels, and also removed sites and individuals with more than 80% missing data and a minor allele frequency (MAF) of <0.01.

### Genetic diversity

We estimated the multilocus lineages (*mll*), and the number of multilocus genotypes (*mlg*), which are the unique combination of alleles across all loci, estimated using package *poppr* (Kamvar, Tabima & Grünwald, 2014) with the R Core Team program V 4.1.2 (R Core Team, 2020). We computed the observed heterozygosity (*H*_*o*_), the expected heterozygosity (*H*_*E*_), and the total heterozygosis (*H*_*T*_), for each SNP locus using *adegenet* V. 2.1.3 (Jombart, 2008; Jombart & Ahmed, 2011) and *hierfstat* (Goudet, 2005). We tested for statistical differences in genetic diversity, with a Bartlett’s and Wilcoxon tests, between young and adults, and among localities, with the R Core Team program V 4.1.2 (R Core Team, 2020).

Additionally, we determined the multilocus heterozygosity (*MLH*)—defined as the total number of heterozygous loci in an individual divided by the number of loci typed in the focal individual—and the standardized multilocus heterozygosity (*sMLH*) for each individual—defined as the number of total heterozygous loci in an individual, divided by the sum of the average observed heterozygosity in the population over the subset of loci successfully typed (Coltman et al., 1999)—using *inbreedR* packages (Stoffel et al., 2016). In the case of genomic data, these estimates are primarily helpful for low-density datasets, where it is unclear whether genotyped markers represent genome-wide diversity or inbreeding (Stoffel et al., 2016).

### Inbreeding

We estimated Wright’s *F*_IS_ statistics in the complete data set with *adegenet* and *hierfstat*. Subsequently, we used Plink v1.9 (Purcell et al., 2007) to estimate the inbreeding index *f* (—*het*), a measure of heterozygosity on a per-individual basis and computes observed and expected autosomal homozygous genotype counts for each sample. We used (*—ibc*) from Plink v1.9 (Purcell et al., 2007), to obtain *Fhat3*, based on the correlation between uniting gametes, which is a measure of inbreeding using allele frequencies in the current population (Keller, Visscher & Goddard, 2011; Yang et al., 2011); these calculations do not take *LD* into account (Purcell et al., 2007). Wilcoxon tests were then used to determine the significant differences in the inbreeding coefficient among the localities.

### Population genetic structure and recent gene flow

To infer patterns of genetic structure, we used different approaches. First, we estimated Edward’s distances (Euclidean) (Edwards, 1971) from the gene frequencies, considering juveniles as a different population, and we obtained an UPGMA dendrogram. Second, we estimated the average paired *F*_*ST*_ using *StAMPP* package (Pembleton, Cogan & Forster, 2013), and we also constructed a matrix of genetic distances among populations, with Nei’s genetic distances (Nei, 1972) using R (packages *hierfstat*). Nei’s paired genetic distances were visualized using a heatmap. Finally, an analysis of individual ancestry by maximum likelihood was performed using ADMIXTURE v.1.23 (Alexander, Novembre & Lange, 2009; Alexander & Lange, 2011), where we tested the number of clusters or *K-values* from 1 to 10, with three different runs using the predetermined parameters. We performed a cross-validation test to determine the best *K-value*.

Recent gene flow (*i.e*., over the last two generations) was inferred using BayesAss V. 3.0.4 (Mussmann et al., 2019). This algorithm uses a probability distribution to decide if newly proposed values will be accepted or rejected for each MCMC sample. The analysis was performed with 50,000,000 iterations, sampling every 1,000 iterations with a burn-in of 5,000,000. We tested several values of acceptance until we determined the final values for alleles frequencies (0.9), migration (0.7), and inbreeding (0.3). We analyzed the convergence of the MCMC with the trace file for each run using Tracer v.17.2.

## Results

### Sequencing and genotyping

The RADseq strategy on 96 *A. tequilana* samples resulted in 39.66 Gb of raw data. The mean quality score (Phred score) was 35.36, and the guanine-cytosine (GC) contents ranged from 49–50%. After demultiplex and removing adapters, the number of reads was 264, 006, 277. Due to the low number of reads in eight samples (JT-6, JT-7, JT-2, JT-10, JT-12, JT-18, JT-28, Ar5-2016), they were excluded from further analysis. Therefore, a total of 88 samples were analyzed. Initially, using a *reference* transcriptome assembly method with Ipyrad, 84, 635 variants were called. After quality control, with depth, allelic number, MAF, and missing data, we retained 979 biallelic SNPs (for a total of 1,958 alleles).

### Genetic diversity

Using multilocus lineage (*mll*) and genotype (*mlg*) analyses, we found that the 88 analyzed plants had different genotypes, *i.e*., each plant presented an unique combination of alleles across all the studied loci. The locality with the highest number of alleles (Table 2) was Alteña (1773), followed by juveniles from Tototlán (J-Tototlán; 1636) and Arenal (1558); while Tototlán (1530) had the lowest number of alleles. The average genetic diversity for all samples of *A. tequilana* was *H*_*T*_ = 0.120, the average observed heterozygosity in all the data set was *H*_*O*_ = 0.129 (SD = 0.177) and the average of expected heterozygosity *H*_*E*_ = 0.120 (SD = 0.149) (Table 2). Observed heterozygosity was significantly higher than expected (*Bartlett’s K-squared* = 12.093, *p-value* = 0.0005), indicating an excess of heterozygous individuals.

**Table 2:** Genetic diversity estimated using 979 SNPs in *Agave tequilana*.

Diversity index	Locality				Full data set
	Alteña	Arenal	Tototlán	Juveniles Tototlán	
Alleles	1,773	1,558	1,530	1,636	1,958
*mlg*	38	19	10	21	88
*mll*	38	19	10	21	88
*H_O_*	0.116 (0.152)	0.127 (0.188)	0.148 (0.197)	0.124 (0.166)	0.129 (0.177)
*H_E_*	0.121 (0.146)	0.109 (0.150)	0.129 (0.154)	0.118 (0.146)	0.120 (0.149)
sMLH	0.921 (0.189)	1.017 (0.265)	1.204 (0.332)	1.005 (0.143)	0.994 (0.231)
MLH	0.113 (0.022)	0.124 (0.034)	0.146 (0.042)	0.123 (0.018)	0.122 (0.028)
f	−0.116 (0.288)	−0.074 (0.153)	0.050 (0.116)	0.075 (0.195)	−0.042 (0.239)
Fhat3	−0.060 (0.043)	−0.055 (0.021)	−0.041 (0.016)	0.229 (0.274)	0.012 (0.182)
*N*	38	19	10	21	88

**Note:**

*mlg*, multilocus genotype; *mll*, multilocus lineage; *H_O_*, observed heterozygosity; *H_E_*, expected heterozygosity; sMLH, standardized multi locus heterozygosity; MLH, multi locus heterozygosity; f, Inbreeding coefficient, measure of heterozygosity on a per-individual basis; Fhat3, inbreeding using allele frequencies; SD in parenthesis; *N*, number of individuals per locality.

Tototlán showed the highest average expected heterozygosity (*H*_*E*_ = 0.129, SD = 0.154), followed by Alteña (*H*_*E*_ = 0.121, SD = 0.146) and J-Tototlán (JT: *H*_*E*_ = 0.118, SD = 0.146), while the population with less genetic diversity was Arenal (*H*_*E*_ = 0.109, SD = 0.150) (Table 2). We found significant differences in the expected heterozygosity (Table S1) between Arenal *vs* Alteña (*Wilcoxon test, p* = 0.000001, *p.adj* = 8.1e−6), Arenal *vs* Tototlán (*Wilcoxon test, p* = 0.00885, *p.adj* = 2.7e−2) and Arenal *vs* J-Tototlán (*Wilcoxon test, p* = 0.043, *p.adj* = 8.6e−2).

**Figure 2:**
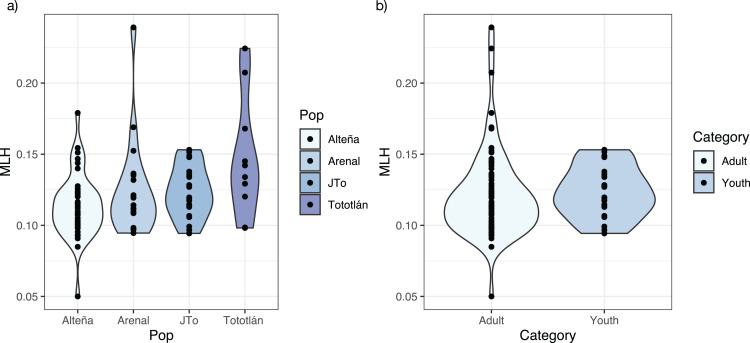
Multilocus heterozygosity, estimated with 979 SNPs, per locality of *A. tequilana*. (A) Individual multilocus heterozygosity per locality; (B) multilocus heterozygosity in adults and juveniles.

The average standardized multi locus heterozygosity was *sMLH* = 0.994 (SD = 0.231) (Table 2), with significant differences in *sMLH* between Alteña *vs* Tototlán (*Wilcoxon test, p* = 0.007, *p.adj* = 0.047). The multi locus heterozygosity (*MLH*) was (*MLH* = 0.122, SD = 0.028), being the highest in Tototlán, followed by Arenal, J-Tototlán, and Alteña (Table 2; Fig. 2A). We found significant differences in *MLH* between Alteña *vs* Tototlán (Table S1; *Wilcoxon test, p* = 0.018, *p.adj* = 0.11).

When we compared the levels of genomic multi locus heterozygosity (*MLH*) in all the adults *vs* juveniles (Fig. 2B), we obtained a higher *MLH* in J-Tototlán (*MLH* = 0.123, SD = 0.018) than in all the adults (*MLH* = 0.121, SD = 0.031) (Table S1; Fig. 2B), but the difference was not significant (*MLH*: *Wilcoxon test, p* = 0.311, *p.adj* = 0.31).

### Inbreeding

**Figure 3:**
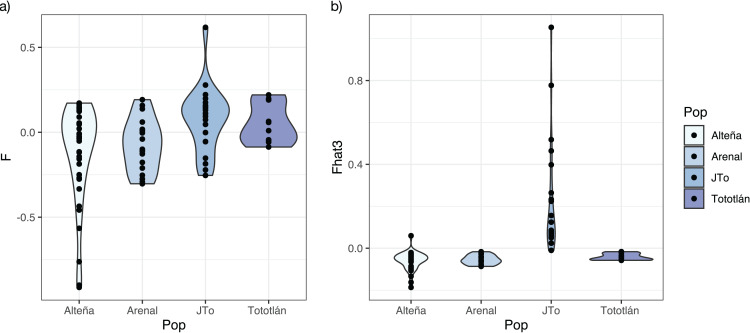
Inbreeding index estimated with 979 SNPs in *A. tequilana*. (A) Coefficient *f* of inbreeding for each population. (B) Fhat3 index.

The average inbreeding coefficient (*F*_*IS*_) in all the analyzed plants of *A. tequilana*, was slightly negative *(F*_*IS*_ = −0.025, SD = 0.218). On the other hand, the average *f* index was negative in the adults from the localities of Alteña, and Arenal, indicating an excess of heterozygotes (*f* = −0.116, *f* = −0.074, respectively) in relation to what would be expected under random mating. In contrast, the juveniles from Tototlán and the adults from the same locality had a positive and moderate level of *f* (*f* = 0.075, *f* = 0.050, respectively) (Table 2, Fig. 3A), indicating a deficit of heterozygotes in these localities.

Based on the genome-wide *Fhat3* inbreeding index, we found that *A. tequilana* individuals have low levels of inbreeding (Fig. 3B), with an average value *Fhat3* = 0.012 (SD = 0.182). Interestingly, while in general adults presented negative values, the juveniles from Tototlán (J-Tototlán) had a positive value (average *Fhat3* = 0.229). A Wilcoxon test showed that the difference in the inbreeding coefficient (*Fhat3*) was significant between young and adults (all adult samples combined) (Table S1, Fig. S1).

### Population genetic structure and recent gene flow

**Figure 4:**
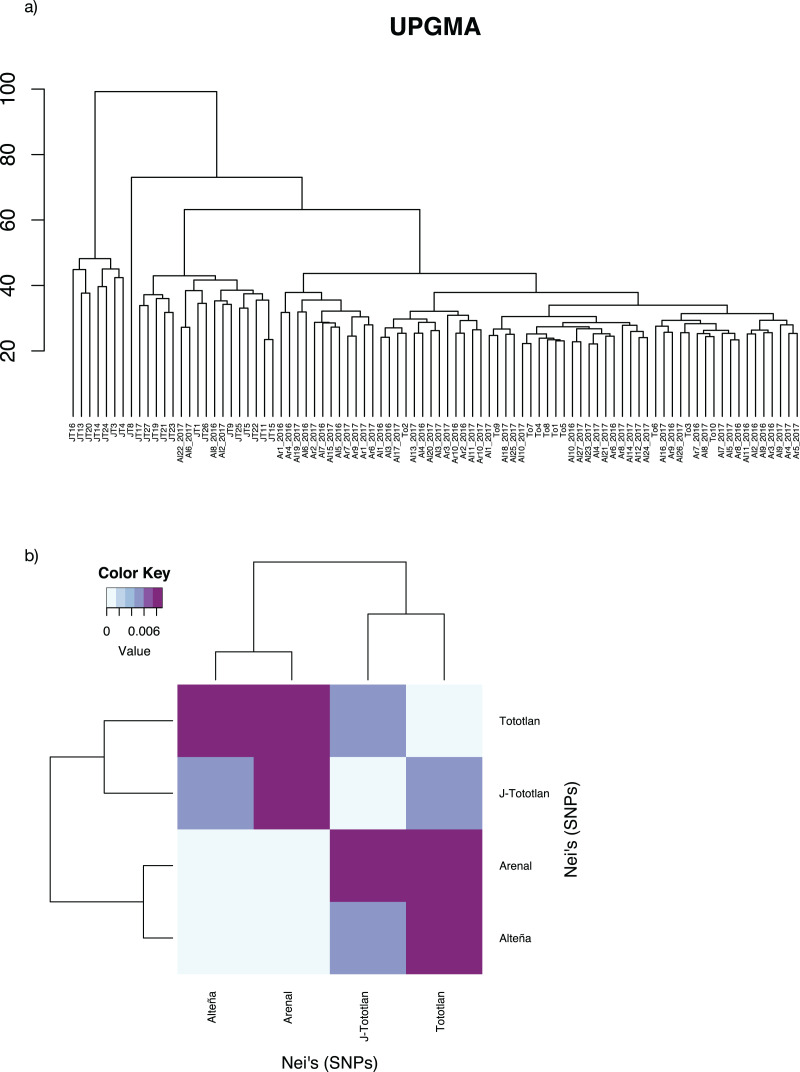
Genetic distances in *Agave tequilana*. (A) UPGMA. (B) Paired Nei’s genetic distance.

The UPGMA analysis, based on Edward’s distance (Fig. 4A), showed different groups. The most divergent group included some juvenile individuals from J-Tototlán (JT16, JT13, JT20 JT14, JT24, JT3, JT4). The largest group was divided into several subgroups and contained the remaining samples of juveniles and adults from Tototlán, Arenal and Alteña.

Low genetic differentiation was found, with an average paired *F*_*ST*_ = 0.0044. The lowest value was found between Arenal and Alteña (*F*_*ST*_ = 0.00009; *p-value* = 0.59), followed by Alteña *vs* J-Tototlán (*F*_*ST*_ = 0.0043; *p-value* = 0.0), Tototlán *vs* J-Tototlán (*F*_*ST*_ = 0.0050; *p-value* = 0.04), Tototlán *vs* Alteña (*F*_*ST*_ = 0.0073; *p-value* = 0.04), Arenal *vs* J-Tototlán (*F*_*ST*_ = 0.0078; *p-value* = 0.0), and the maximum value was found between Tototlán and Arenal (*F*_*ST*_ = 0.0101; *p-value* = 0.0). Similar results were obtained using Nei’s distance, which ranged from 0.0010 to 0.0089 (average Nei’s genetic distance = 0.0060) (Fig. 4B).

According to the ADMIXTURE analysis with three independent runs, and different values of *K* (from 1 to 10), the cross-validation error estimates showed that the best model fit was *K* = 1 (CV = 0.26261) (Fig. S2). Nevertheless, we plotted the values from *K* = 2 to *K* = 6 to explore for genetic structure within samples (Fig. S3). We found that all populations shared alleles, without a clear differentiation or structure among localities.

**Figure 5:**
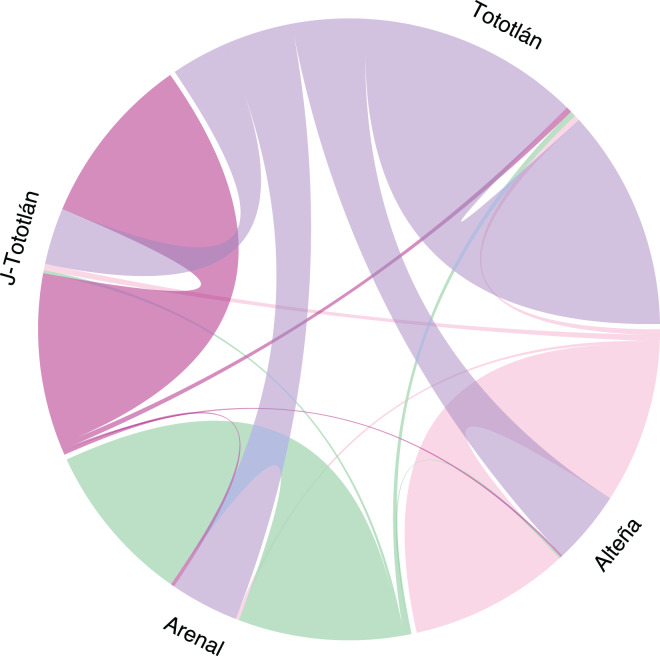
Recent migration in *Agave tequilana*. Migration rates estimated using BayesAss V. 3.0.4 (BA3-SNPs) with 979 SNPs. The population of Tototlán is represented by color purple, Alteña: light pink, Arenal: green, J-Tototlán: pink. Proportion of migrants and the direction, is represented by the colored lines, being thicker where the migration rate is higher.

The analysis of recent migration rates using BayesAss software suggested a high migration rate (*m*) from the source population of Tototlán (Table S2, Fig. 5). Gene flow varied from 0.007 to 0.309 (average 0.25) between pairs of localities. We found that the highest inferred migration rate (Fig. 5) was from Tototlán (color purple) to Alteña (light pink), with *m* = 0.309 (SD = 0.013); thus a fraction of individuals in Alteña were migrants derived from Tototlán; followed by Tototlán to Arenal (green), *m* = 0.289 (SD = 0.023) and Tototlán to J-Tototlán (pink) *m* = 0.238 (SD = 0.029). In comparison, the lowest migration rate was from Alteña to Arenal *m* = 0.007 (SD = 0.007).

## Discussion

The new sequencing technologies are now routinely used to discover a large number of single nucleotide polymorphisms (SNPs) (Elshire et al., 2011; Barrera-Redondo, Piñero & Eguiarte, 2020; Eguiarte et al., 2022; Klimova et al., 2022). These new technologies have been particularly useful for plant species with complex and large genomes, such as agaves (Eguiarte et al., 2013, 2021). Our work represents the first report of genetic diversity and differentiation patterns based on genome-wide SNPs in *A. tequilana*, a species of substantial economic value.

We found that the GC content in *A. tequilana* is higher (>49%) than in other monocots (33–48%) (Šmarda et al., 2014). In theory, a higher GC base pair content in a genome provides higher thermal stability than AT base content (Šmarda et al., 2014). It has been documented that richer content of GC in plants is related to a greater tolerance to extreme temperatures and it was also suggested that it facilitates complex gene regulation (Šmarda et al., 2014).

### Genetic diversity

Cultivated *Agaves* appear to have lower genetic variation in comparison to their wild relatives (Eguiarte et al., 2013, 2021; Félix-Valdez et al., 2016; Figueredo-Urbina, Casas & Torres-García, 2017; Trejo et al., 2018; Cabrera-Toledo et al., 2020, 2022), mainly due to human management, artificial selection, incipient domestication, and vegetative propagation. We found that the expected heterozygosity in all the samples in *A. tequilana* was *H*_*E*_ = 0.120. We also found higher expected heterozygosity in juveniles in comparison to adults, perhaps due to the gene flow with other *Agav*e populations, (see below in the *Low population structure and recent gene flow* section).

To compare our data, we can mention *A. angustifolia* in wild and cultivated plants used to produce an alcoholic drink similar to tequila (bacanora), using SNPs derived from restriction site associated DNA sequencing, where Klimova et al. (2022) detected a *H*_*E*_ = 0.25. Similar results were also obtained from other Agavoideae, genotyped with next-RAD strategies, as *H*_*E*_ of 0.173 and 0.249 were reported for *Yucca valida* and *Yucca capensis*, respectively (Arteaga, Bello-Bedoy & Gasca-Pineda, 2020).

Previous genetic studies on *A. tequilana* have reported a broad range of genetic diversity estimates, but we must point out that they used very different molecular methods, not SNP based analysis. For instance, the highest expected heterozygosity *H*_*E*_ = 0.205 was reported using AFLPs by Rivera-Lugo, García-Mendoza & Simpson (2018), although the sample size was very small, (only five plants from a locality in the state of Guanajuato). In an ISSRs based study of 22 plants collected at Tequila, Jalisco, Vargas-Ponce et al. (2009) reported a *H*_*E*_ = 0.118, similar to what we estimated in the present study (*H*_*E*_ = 0.120). In contrast, based on microsatellites (with eight loci), Trejo et al. (2018), analyzing 23 plants sampled in cultivated fields of Tequila from central Jalisco, reported the same genotype in all sampled individuals (*i.e*., *H*_*E*_ = 0). Similar results were obtained with RAPDs markers, where only 1 of 124 RAPD products (0.8%) was polymorphic, and 39 of 40 plants were completely isogenic (Gil-Vega et al., 2001). In other less-intensively managed populations around Tequila town, different levels of genetic diversity have been detected with microsatellites in the varieties *A. tequilana* “Sigüin” *H*_*E*_ was 0.409 and in *A. tequilana* “Chato” *H*_*E*_ was 0.435 (Trejo et al., 2018). However, the comparison among studies is complicated as pointed out above, given the differences in the molecular methodologies and sampling designs.

*Agave tequilana* is a species that has been intensively managed since the beginning of the last century (Trejo et al., 2018). Therefore we decided to compare its diversity to different cultivated species from Mexico using SNPs. For instance, in the common pumpkin (zucchini, *Cucurbita pepo ssp. pepo*) Martínez-González et al. (2021) found a *H*_*E*_ = 0.185 in populations distributed along Mexico using tunable genotyping by sequencing (tGBS), or for the cultivated runner-red bean (*Phaseolus coccineus*), Guerra-García et al. (2017), reported a range in *H*_*E*_ = 0.167 to 0.221 using genotyping by sequencing (GBS), values similar to what we found in *A. tequilana*.

Genetic diversity is necessary for further evolutionary response to natural selection pressures and to allow for crop improvement (Frankham, 2010; Gepts & Hancock, 2006), it enhances resilience to climate change, by providing the traits that are key to the efficiency and adaptability of production systems (Bruford et al., 2017). We observed that genetic diversity, even if low compared with other *Agave* and *Yucc*a populations, is still maintained in the “Bat-friendly” localities in Jalisco.

### Excess of heterozygotes and inbreeding

Inbreeding and excess of heterozygotes are often estimated through Wright’s inbreeding coefficient (*F*_*IS*_) and related estimates, measuring the deviation from Hardy-Weinberg equilibrium (Wright, 1951), which allows us to infer how mating processes and/or different selection regimes are occurring within the population (Hedrick, 2011).

In the adults of *A. tequilana* we estimated an excess of heterozygotes. For instance, there are many examples of clonal propagated highly heterozygous species, such as the date palm (*Phoenix dactylifera* L.) a monocot dioecious species, typically clonally propagated (Hazzouri et al., 2019). Another well-known example is the potato (*Solanum tuberosum* L.), where its high heterozygosity has been explained by the asexual propagation and polyploidy, which provides the potential to display great plasticity that favors adaptation to different environments and challenges (The Potato Genome Sequencing Consortium, 2011; Manrique-Carpintero et al., 2018). We can also mention the cassava (*Manihot esculenta ssp*.) with a wide tropical distribution, a vegetatively propagated crop (Taye, 1998; Santana et al., 2009) and it is highly heterozygous (Fregene et al., 2003; Siqueira et al., 2010; Wang et al., 2014). In cassava it is well documented that long-established clones are highly heterozygous, while plants originating from seeds are characterized by high variance in the degree of inbreeding (Pujol, David & McKey, 2005; McKey et al., 2010). Furthermore, in the domesticated grape (*V. vinifera ssp. sativa*), cultivars are clonally propagated and highly heterozygous but carry many deleterious recessive mutations (Velasco et al., 2007).

In *A. tequilana* we found that the observed heterozygosity values was generally higher than expected, resulting in negative *F*_*IS*_ values, also with the *Fhat3* index the adults presented negative values, while the juveniles from Tototlán (J-Tototlán) had a positive value, apparently due to some inbreeding in this population. Inbreeding in the juveniles may result of few reproductive events in *A. tequilana* in this locality, so there may be self-pollination or crosses among relatives.

Negative *F*_*IS*_ and heterozygosity excess in the adults may have several potential explanations. It may be due to natural and artificial selection by the farmers, that remove small and weak plants (that may be the more homozygous individuals) and select for the most vigorous (and potentially heterozygous plants). A well know case of heterozygote advantage (heterosis) is exhibited in corn, which results from the use of hybrid seeds for agriculture (Hamilton, 2009, page 38). Heterozygote excess should increase over the life cycle either because of progressive selection against deleterious recessive alleles revealed in the homozygous state or by selection favoring individuals bearing differing alleles (Mitton, 1989; Stoeckel et al., 2006). Also, negative *F*_*IS*_ may be a maintained by asexual reproduction (Balloux, Lehmann & de Meeüs, 2003; Alberto et al., 2005; Ruggiero, Reusch & Procaccini, 2005) that preserved heterozygosity or may even increase it by somatic mutation over generations (Judson & Normark, 1996; Welch & Meselson, 2000), as these mutations can accumulate without sexual reproduction to purge it (Klekowski, 1988; Schoen & Schultz, 2019).

Negative *F*_*IS*_ are not uncommon in plants and for instance have been reported in several managed species, such as *Agave angustifolia, A. tequilana* and *A. rhodacantha*, with *F*_*IS*_ ranging from −0.8420 to 0.1326 (Cabrera-Toledo et al., 2022), in the perennial cultivated scarlet runner bean (*Phaseolus coccineus; F*_*IS*_ = −0.159) (Guerra-García et al., 2017), and in long-living species, such as *Astrocaryum mexicanum* (mean for adults *F*_*IS*_ = −0.41 and for seeds *F*_*IS*_ = −0.19) (Eguiarte, Perez-Nasser & Piñero, 1992). Nevertheless, to be certain if there is heterozygote advantage in *A. tequilana*, field experiments and more analyzes are required.

### Low population structure and recent gene flow

Genetic structure results from an interaction among ecological factors, historical events, and evolution processes (Cheng, Kao & Dong, 2020). In natural agave populations, low levels of genetic differentiation and structure among populations have been reported (Eguiarte et al., 2013, 2021), and accordingly, we found very low genetic differentiation (average paired *F*_*ST*_ = 0.0044), and alleles shared among all populations. This low differentiation could be due to intensive management of the species, where propagation mainly occurs by propagules and/or clonal. It can also be accounted to the fact that populations have not been separated for so long, and ancestral polymorphisms are still maintained. The juvenile individuals from Tototlán were slightly more divergent than the rest of the populations, however, they did not show significant differences. In our study we found the lowest reported *F*_*ST*_ value in *Agave*. For instance, in *A. angustifolia* Klimova et al. (2022) found an average paired *F*_*ST*_ = 0.005, while in other *Agave* species Eguiarte et al. (2013) mentions a range of *F*_*ST*_ from 0.057 (in *Agave cocui* with isozymes) to 0.76 (in *Agave parry* cultivated with microsatellites).

Moreover, two of the studied localities (Arandas and Tototlán) are relatively close to each other geographically (~90 km), while the most distant were Arandas and Arenal (~250 km). Gene flow may affect population structure, as Agaves have long-distance pollen dispersal usually conducted by nectar feeding bats, including *Leptonycteris yerbabuenae, L. nivalis*, and *Choeronycteris mexicana* (Molina-Freaner & Eguiarte, 2003; Silva-Montellano & Eguiarte, 2003; Rocha, Valera & Eguiarte, 2005; Sánchez & Medellin, 2007; Trejo-Salazar, Scheinvar & Eguiarte, 2015; Trejo-Salazar et al., 2016).

Gene flow, therefore, may play an important role in the evolution process of populations because it can increase genetic diversity as new alleles are introduced into the new population (Bhandari et al., 2017). Apparently, the main source of origin of gene flow in this study was Tototlán. Also, this locality is the one with the highest genomic diversity (*H*_*E*_ and *H*_*o*_); thus, we consider important to continue monitoring it for a possible source of variation.

### Possible conservation strategies

*Agave tequilana* is one of the most important economic crop in Mexico (Servicio de Información Agroalimentaria y Pesquera, SIAP, 2020). Various strategies have been proposed and implemented to maintain the genetic diversity in agaves, such as the “Bat-Friendly” program, which aims to generate conservation collaboration with tequila and mezcal producers, especially with the smallest and more traditional producers, recognizing them as more ecological friendly companies, since they allow a small percentage of agaves to flower, promoting bat-mediated pollination to recover and to maintain the genetic diversity and, at the same time, generate conditions for healthy ecosystems for bats and agaves (Trejo et al., 2016; batfriendly.org). Nevertheless, in terms of the program, we consider that it is still too early in the game to show its potential benefits to maintain genetic diversity. However, it is important to highlight that in the present study viable seeds were generated by the naturally pollinated inflorescences, so there can be natural population recruitment. We believe that in future generations, once allowing bat pollination in the agave plantations become mainstream, bats and agaves will be able to continue their millennial association.

We also want to emphasize the importance of bat pollination in agaves in general, since the movement of these mammals is closely related to the reproductive success of the plants (Trejo-Salazar et al., 2016). Furthermore, the long-distance pollination and dispersal capabilities of bats provide a favorable mechanism for introducing new alleles, resulting in the maintenance of large effective population sizes, genetic connectivity, and gene flow even in fragmented, cultivated, and semi-managed populations, counteracting the genetic impacts of habitat fragmentation. Therefore, a conservation strategy for the agaves should also include the conservation of its primary pollinators.

In a climate change scenario, it is crucial that populations maintain genetic diversity to be able to adapt to the new climatic conditions. It has also been suggested that the inclusion of different varieties of agave in the same field could serve as a germplasm resource and reduce the risk of pests and the loss of diversity (Álvarez-Ríos et al., 2020). Nevertheless, due to restrictions established in the denomination of origin (DO), published in 1974 (Diario Oficial de la Federación, 1974, 1997), which limits the integration of other varieties of *Agave tequilana* besides the var. azul (*e.g*., the varieties “azul lisado”, “chato”, “bermejo”, “pata de mula”, “sigüin”, “sahuayo”, “moraleño”, “mano larga”, “criollo” and “zopilote”; Colunga-García Marín & Zizumbo-Villarreal, 2007; Trejo et al., 2018) it is difficult for producers to introduce other species or varieties to their plantations. Therefore, it may be necessary to change the DO, where new varieties of agaves would be incorporated; this, in turn, would facilitate the preservation of genetic variation, ecological and cultural diversity of this species (Vargas-Ponce et al., 2009).

## Conclusions

The main objective of this study was to evaluate the levels of genomic variation in three traditionally managed areas of *A. tequilana* in Jalisco, Mexico. We found an excess of heterozygotes in the adults, and lower genomic diversity than in the closely related *A. angustifolia*, but the variation, even if low, was higher than some reports for the species made in more intensive management sites, for example, from around the town of Tequila, Jalisco. We found low genetic differentiation, as reported in most other studies conducted within this genus (Eguiarte et al., 2013, 2021), but in our study it was even lower than in previous studies. We also detected recent gene flow among populations.

The relatively high levels of observed heterozygosity of *A. tequilana* found in the adults in our study maybe be explained by more traditional management and clonal propagation. Also, occasional sexual reproduction, and exchange of seeds or propagules by producers may play an important role in maintaining diversity in *A. tequilana*.

Our study also demonstrated that massive sequencing related genomic strategies using SNPs, along with the studies of Cabrera-Toledo et al. (2022) and Klimova et al. (2022), will allow to gather good comparative data for the future conservation and management of this important genus and for the study of its evolutionary processes, including both wild and cultivated species.

## Supplemental Information

10.7717/peerj.14398/supp-1 Supplemental Information 1 Wilcoxon test performed for all the diversity and inbreeding coefficients.

10.7717/peerj.14398/supp-2 Supplemental Information 2 Migration rates estimated by BayesAss V.3.0.4 (BA3-SNPs).

10.7717/peerj.14398/supp-3 Supplemental Information 3 Compare of inbreeding index, with 979 SNPs, between adults (reproduced by propagules) *vs*. juveniles (reproduced by seed). (a) Coefficient *f* of inbreeding by population. (b) *Fhat3*.

10.7717/peerj.14398/supp-4 Supplemental Information 4 Plot of Admixture cross validation error from K = 1 through K = 10, in *A. tequilana* of Jalisco, Mexico.

10.7717/peerj.14398/supp-5 Supplemental Information 5 Admixture analysis for (a) K = 2, (b) K = 3, (c) K = 4, (d) K = 5, (e) K = 6, showing the assignment probability of individuals from *Agave tequilana*.

## Additional Information and Declarations

### DNA Deposition

The following information was supplied regarding the deposition of DNA sequences: The raw data is available at Genbank Sequence Read Archive (SRA): SRR20218765.

### Data Availability

The following information was supplied regarding data availability: The raw vcf file is available at Zenodo: Ruiz Mondragon Karen Yazmin, Aguirre Planter Erika, Gasca-Pineda Jaime, Klimova Anastasia, Trejo-Salazar Roberto, Reyes Guerra Marco Antonio, Medellín Rodrigo, Piñero Daniel, Lira Saade Rafael, & Eguiarte Luis. (2023). Conservation genomics of *Agave tequilana* Weber var. azul: low genetic differentiation and heterozygote excess in the tequila agave from Jalisco, Mexico. https://doi.org/10.5281/zenodo.7041493.
