# Supplementary material for: Conservation genomics of Agave tequilana Weber var. azul: low genetic differentiation and heterozygote excess in the tequila agave from Jalisco, Mexico
Source: PeerJ. 2022 Nov 17;10:e14398. doi: 10.7717/peerj.14398 (PMC9676017; doi:10.7717/peerj.14398)
Supplement: Supplemental Information 1 [file peerj-10-14398-s001.docx]

| ***Ho vs HS*** | ***Group 1*** | ***Group 2*** | ***p*** | ***p.signif*** | ***method*** |
| --- | --- | --- | --- | --- | --- |
| ***1*** | Arenal | Alteña | 0.00000135 | **** | Wilcoxon |
| ***2*** | Arenal | Tototlan | 0.00885 | ** | Wilcoxon |
| ***3*** | Arenal | J-Tototlan | 0.0431 | * | Wilcoxon |
| ***4*** | Alteña | Tototlan | 0.135 | ns | Wilcoxon |
| ***5*** | Alteña | J-Tototlan | 0.151 | ns | Wilcoxon |
| ***6*** | Tototlan | J-Tototlan | 0.516 | ns | Wilcoxon |
| ***MLH*** | | | | | |
| ***1*** | Alteña | Arenal | 0.435 | ns | Wilcoxon |
| ***2*** | Alteña | JTo | 0.0722 | ns | Wilcoxon |
| ***3*** | Alteña | Tototlán | 0.0182 | * | Wilcoxon |
| ***4*** | Arenal | JTo | 0.503 | ns | Wilcoxon |
| ***5*** | Arenal | Tototlán | 0.0854 | ns | Wilcoxon |
| ***6*** | JTo | Tototlán | 0.139 | ns | Wilcoxon |
| ***sMLH*** | | | | | |
| ***1*** | Alteña | Arenal | 0.285 | ns | Wilcoxon |
| ***2*** | Alteña | JTo | 0.0539 | ns | Wilcoxon |
| ***3*** | Alteña | Tototlán | 0.00775 | ** | Wilcoxon |
| ***4*** | Arenal | JTo | 0.63 | ns | Wilcoxon |
| ***5*** | Arenal | Tototlán | 0.0694 | ns | Wilcoxon |
| ***6*** | JTo | Tototlán | 0.0955 | ns | Wilcoxon |
| ***Fhat3*** | | | | | |
| ***1*** | Alteña | Arenal | 0.927 | ns | Wilcoxon |
| ***2*** | Alteña | JTo | 1.16E-14 | **** | Wilcoxon |
| ***3*** | Alteña | Tototlán | 0.139 | ns | Wilcoxon |
| ***4*** | Arenal | JTo | 1.52E-11 | **** | Wilcoxon |
| ***5*** | Arenal | Tototlán | 0.0559 | ns | Wilcoxon |
| ***6*** | JTo | Tototlán | 4.51E-08 | **** | Wilcoxon |
| ***f*** | | | | | |
| ***1*** | Alteña | Arenal | 0.86 | ns | Wilcoxon |
| ***2*** | Alteña | JTo | 0.013 | * | Wilcoxon |
| ***3*** | Alteña | Tototlán | 0.108 | ns | Wilcoxon |
| ***4*** | Arenal | JTo | 0.0136 | * | Wilcoxon |
| ***5*** | Arenal | Tototlán | 0.0396 | * | Wilcoxon |
| ***6*** | JTo | Tototlán | 0.693 | ns | Wilcoxon |
| ***Adults vs Youth*** | | | | | |
| ***MLH*** | Adult | Youth | 0.311 | ns | Wilcoxon |
| ***sMLH*** | Adult | Youth | 0.328 | ns | Wilcoxon |
| ***F*** | Adult | Youth | 0.0122 | * | Wilcoxon |
| ***Fhat3*** | Adult | Youth | 8.91E-12 | **** | Wilcoxon |
