# Supplementary material for: Conservation genomics of Agave tequilana Weber var. azul: low genetic differentiation and heterozygote excess in the tequila agave from Jalisco, Mexico
Source: PeerJ. 2022 Nov 17;10:e14398. doi: 10.7717/peerj.14398 (PMC9676017; doi:10.7717/peerj.14398)
Supplement: Supplemental Information 2 [file peerj-10-14398-s002.docx]

| ***Pop1*** | ***Pop2*** | ***Migration rate*** | ***SD*** |
| --- | --- | --- | --- |
| Alteña | Alteña | 0.6746 | 0.0077 |
| Alteña | Arenal | 0.0146 | 0.014 |
| Alteña | J-Tototlán | 0.0267 | 0.0178 |
| Alteña | Tototlán | 0.0238 | 0.0222 |
| Arenal | Alteña | 0.0079 | 0.0077 |
| Arenal | Arenal | 0.6813 | 0.0139 |
| Arenal | J-Tototlán | 0.0133 | 0.0128 |
| Arenal | Tototlán | 0.0238 | 0.022 |
| J-Tototlán | Alteña | 0.0079 | 0.0077 |
| J-Tototlán | Arenal | 0.0145 | 0.0139 |
| J-Tototlán | J-Tototlán | 0.7215 | 0.0246 |
| J-Tototlán | Tototlán | 0.0237 | 0.0221 |
| Tototlán | Alteña | 0.3096 | 0.0131 |
| Tototlán | Arenal | 0.2896 | 0.0231 |
| Tototlán | J-Tototlán | 0.2385 | 0.0297 |
| Tototlán | Tototlán | 0.9287 | 0.0351 |
