# Supplementary figures and images for: Conservation genomics of Agave tequilana Weber var. azul: low genetic differentiation and heterozygote excess in the tequila agave from Jalisco, Mexico
Source: PeerJ. 2022 Nov 17;10:e14398. doi: 10.7717/peerj.14398 (PMC9676017; doi:10.7717/peerj.14398)

a)

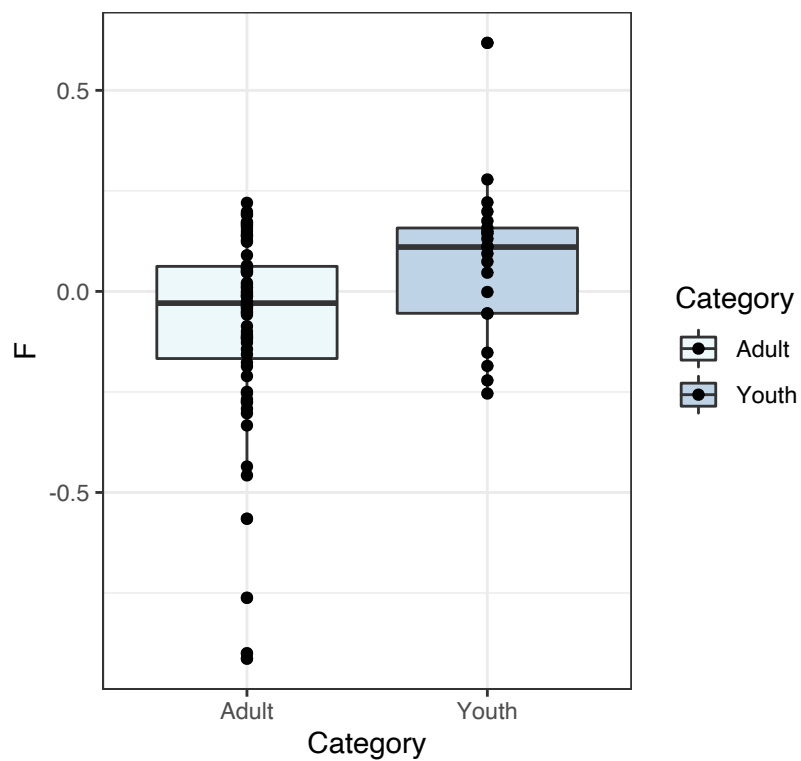

b)

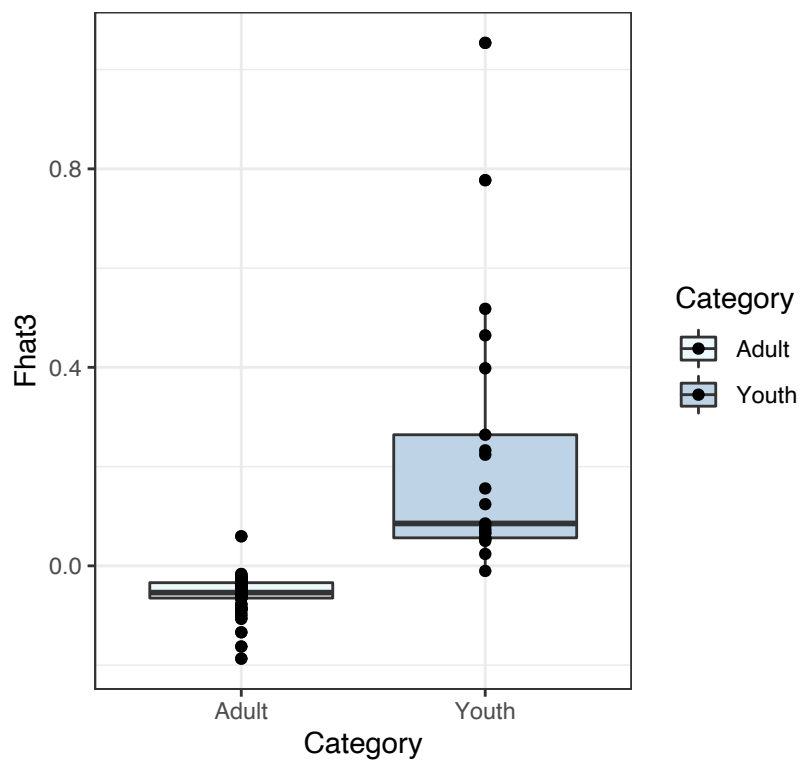

Supplement: Supplemental Information 3 [file peerj-10-14398-s003.pdf]

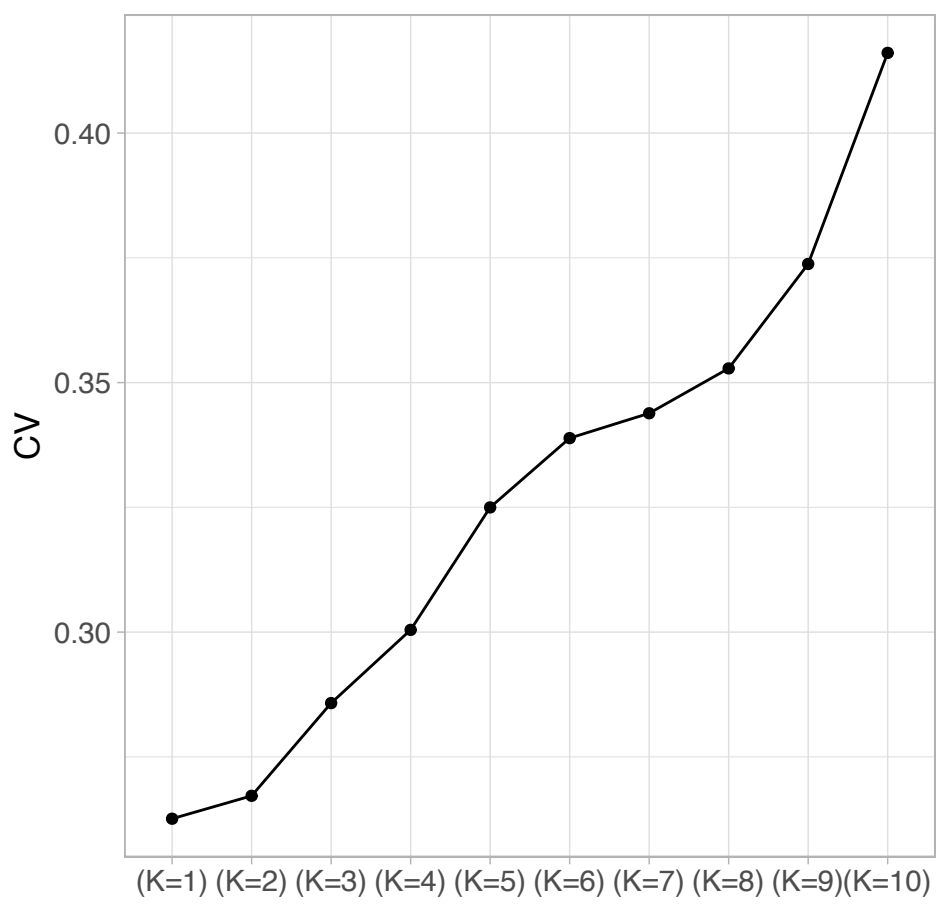

Supplement: Supplemental Information 4 [file peerj-10-14398-s004.pdf]

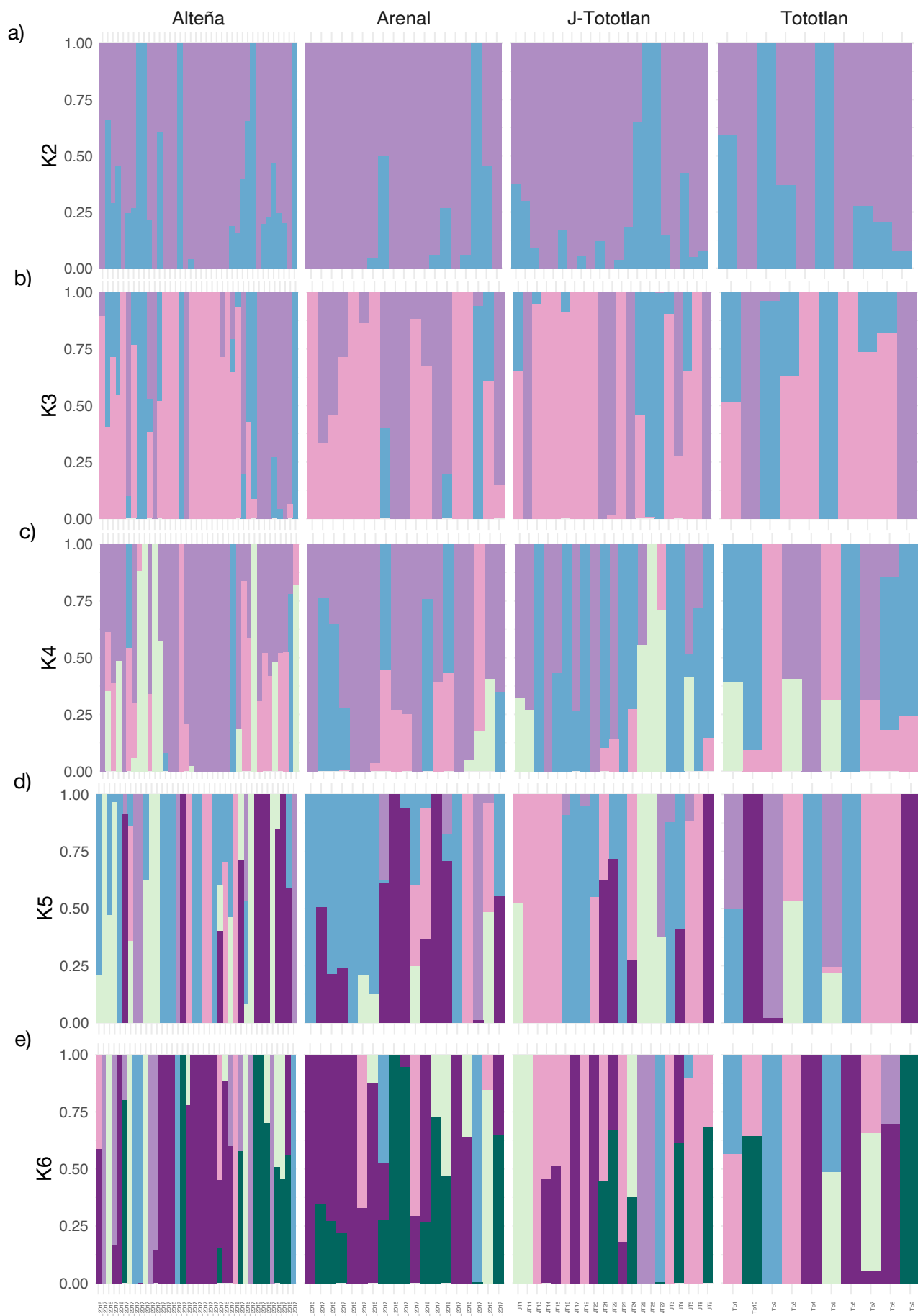

Supplement: Supplemental Information 5 [file peerj-10-14398-s005.pdf]
